# Supplementary material for: A Humanized Yeast Phenomic Model of Deoxycytidine Kinase to Predict Genetic Buffering of Nucleoside Analog Cytotoxicity
Source: Genes (Basel). 2019 Sep 30;10(10):770. doi: 10.3390/genes10100770 (PMC6826991; doi:10.3390/genes10100770)
Supplement: Supplementary file 1 [file genes-10-00770-s001.zip › Additional_Files/Additional_File7_GOTermHeatmaps.zip!/Additional_File7_GOTermHeatmaps/adenyl-nucleotide_exchange_factor_activity.pdf]

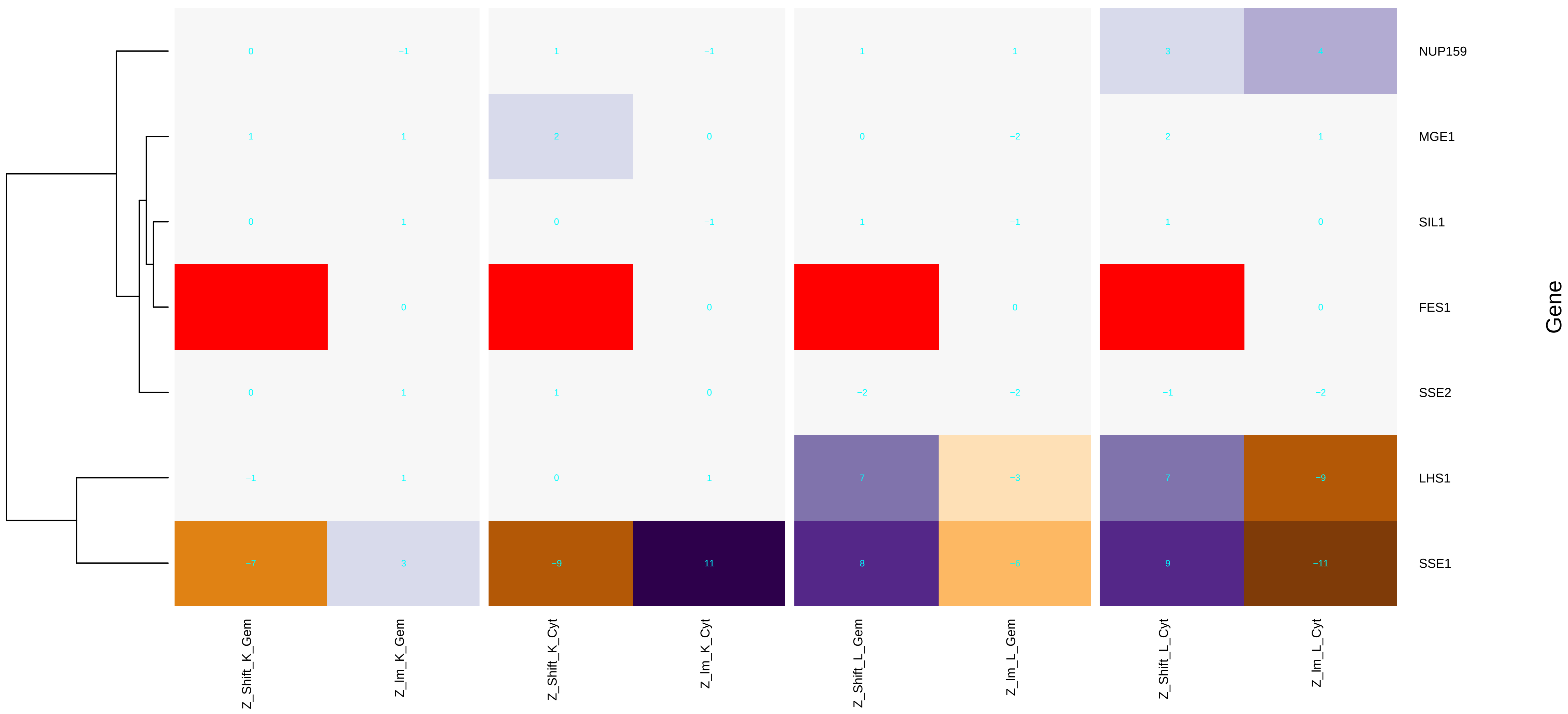

NUP159
MGE1
SIL1
FES1
SSE2
LHS1
SSE1
Gene
Z_Shift_K_Gem
Z_Im_K_Gem
Z_Shift_K_Cyt
Z_Im_K_Cyt
Z_Shift_L_Gem
Z_Im_L_Gem
Z_Shift_L_Cyt
Z_Im_L_Cyt
0 −1 1 −1 1 1 3 4
1 1 2 0 0 −2 2 1
0 1 0 −1 1 −1 1 0
0 0 0 0
0 1 1 0 −2 −2 −1 −2
−1 1 0 1 7 −3 7 −9
−7 3 −9 11 8 −6 9 −11
